# Development and Validation of a Prediction Model to Estimate Individual Risk of Pancreatic Cancer

**DOI:** 10.1371/journal.pone.0146473

**Published:** 2016-01-11

**Authors:** Ami Yu, Sang Myung Woo, Jungnam Joo, Hye-Ryung Yang, Woo Jin Lee, Sang-Jae Park, Byung-Ho Nam

**Affiliations:** 1 Biometric Research Branch, Division of Cancer Epidemiology and Prevention, Research Institute & Hospital, National Cancer Center, Goyang-si, Gyeonggi-do, Republic of Korea; 2 Center for Liver Cancer, Research Institute & Hospital, National Cancer Center, Goyang-si, Gyeonggi-do, Republic of Korea; Indiana University School of Medicine, UNITED STATES

## Abstract

**Introduction:**

There is no reliable screening tool to identify people with high risk of developing pancreatic cancer even though pancreatic cancer represents the fifth-leading cause of cancer-related death in Korea. The goal of this study was to develop an individualized risk prediction model that can be used to screen for asymptomatic pancreatic cancer in Korean men and women.

**Materials and Methods:**

Gender-specific risk prediction models for pancreatic cancer were developed using the Cox proportional hazards model based on an 8-year follow-up of a cohort study of 1,289,933 men and 557,701 women in Korea who had biennial examinations in 1996–1997. The performance of the models was evaluated with respect to their discrimination and calibration ability based on the C-statistic and Hosmer-Lemeshow type χ^2^ statistic.

**Results:**

A total of 1,634 (0.13%) men and 561 (0.10%) women were newly diagnosed with pancreatic cancer. Age, height, BMI, fasting glucose, urine glucose, smoking, and age at smoking initiation were included in the risk prediction model for men. Height, BMI, fasting glucose, urine glucose, smoking, and drinking habit were included in the risk prediction model for women. Smoking was the most significant risk factor for developing pancreatic cancer in both men and women. The risk prediction model exhibited good discrimination and calibration ability, and in external validation it had excellent prediction ability.

**Conclusion:**

Gender-specific risk prediction models for pancreatic cancer were developed and validated for the first time. The prediction models will be a useful tool for detecting high-risk individuals who may benefit from increased surveillance for pancreatic cancer.

## Introduction

Pancreatic cancer represents the fifth-leading cause of cancer-related death in Korea and the seventh worldwide. It has a dismal 5 year survival rate of 7.6% in Korea [1], mainly due to unresectable disease in 80–90% of patients at the time of diagnosis [2]. Pancreatic cancer patients seldom exhibit disease-specific symptoms until late in the course of disease progression, and the impact of standard therapy is limited. Despite advances in the screening and early detection of other cancers, such as gastric cancer and breast cancer, no reliable screening tool exists for pancreatic cancer. Because of the relatively low incidence of the disease, current efforts are only focused on early detection and screening in patients at high risk of developing the disease.

A screening strategy has not been established for sporadic pancreatic cancer. Because pancreatic cancer-specific symptoms occur late in the course of the disease, early detection will require screening asymptomatic individuals. Invasive pancreatic cancer develops from precancerous non-invasive precursor lesions called pancreatic intraepithelial neoplasia (PanIN), which progresses from PanIN1 to PanIN3 (carcinoma in situ) [3]. However, the timeline of the progression of pancreatic cancer is not well established. In a case series, Brat and colleagues reported the presence of PanIN 17 months to 10 years before the clinical diagnosis of cancer [4]. Two major obstacles restrict our ability to screen for pancreatic cancer: an absence of a high-risk group of patients and the absence of sensitive and specific marker(s) for detecting early stages of pancreatic cancer. However, even if a biomarker with very high sensitivity and specificity is identified, screening the general population for asymptomatic pancreatic cancer would not be cost effective or practical. Thus, screening for asymptomatic pancreatic cancer will likely require filtering the population into at least two sequential groups in order to enrich the population and allow cost-effective screening [5]. The first filter could be the selection of a high-risk group (i.e., a population of individuals at a higher than average risk of pancreatic cancer [6]), and the second filter could be to identify individuals with a unique clinical phenotype using one or more biomarker(s) of early stage pancreatic cancer or non-invasive imaging [5, 7]. Currently, individuals with genetic syndromes that are associated with a high incidence of pancreatic cancer and those who have at least two first-degree relatives with pancreatic cancer are screened by endoscopic ultrasonography [8]. However, these patients account for less than 5% of all pancreatic cancer cases. An entirely different approach should be developed for screening sporadic pancreatic cancer.

The present study is the largest population-based cohort study of pancreatic cancer to date in Korea and provides a unique opportunity to develop a model for predicting the individual risk of developing pancreatic cancer.

## Materials and Methods

### Study population

The study population consisted of men and women who participated in a biennial health examination conducted by Korean Health Insurance Corporation, including government employees, schoolteachers, company employees, and their dependents, between 1996 and 1997. A total of 1,289,933 men and 557,701 women aged 30 to 80 years who had no history of any cancer at baseline and during the first two years of follow-up, without any missing values for the primary risk factors (age, height, body mass index (BMI), fasting glucose, urine glucose, cholesterol, smoking, age at smoking initiation, meal preference, frequency of meat consumption, eating habits), were included in the model development.

To assess the performance of the models, we used an independent population medically evaluated by the National Health Insurance Corporation between 1998 and 1999 that was free of any cancer at baseline as a validation cohort. A total of 500,046 men and 627,629 women were included in the validation data set.

The participants were followed from the date of the health examination until December 31, 2007, and the event was defined as the first diagnosis of pancreatic cancer (median follow-up time: men, 11.49 years; women, 10.72 years in the development set and men, 8.50 years; women, 8.46 years in the validation set). Individuals who did not develop cancer until the end of the follow-up were censored.

This study was approved by the Institutional Review Board of the National Cancer Center, Korea (IRB no. NCCNCS09-305). The need for participant consent was waived by the ethics committee because this study involved routinely collected medical data that were anonymously managed at all stages, including data cleaning and statistical analysis.

### Data collection

The incidence of pancreatic cancer among participants up to December 31, 2007, was identified through the Korean Central Cancer Registry (KCCR) database. The incidence of pancreatic cancer was classified according to ICD-10 codes (C25) [9]. Deaths, including cancer deaths, were identified from the death records of the National Statistical Office and National Health Insurance Corporation. During the health examination, participants responded to a questionnaire about previous disease history, eating habits, meal preferences, frequency of meat intake, drinking habits, amount of alcohol consumed at a time, duration of smoking, amount of smoking per day, year of smoking cessation, and number of times per week they participated in physical activity. Height, weight, systolic and diastolic blood pressure, total cholesterol, and fasting blood and urine glucose levels were measured directly. BMI was calculated as the weight in kilograms divided by the square of the height in meters. The age at smoking initiation was calculated from the duration of smoking and age at baseline.

### Prediction model development

The Cox proportional hazard model was used to develop gender-specific risk prediction models. Significant risk factors for pancreatic cancer were identified by crude and age-adjusted Cox regression. The time to event was defined as the time between the date of the health examination and the date of the first diagnosis of pancreatic cancer. Potential risk factors considered in the analyses included previous disease history (hepatitis, diabetes, and any other cancer), eating habits (bland, moderate, spicy, or salty), meal preference (meat vs. vegetables), frequency of meat intake (≤1 time/week, 2–3 times/week, or ≥4 times/week), drinking habit (≤2–3 times/month or ≥1–2 times/week), amount of alcohol consumed at a time, duration of smoking, amount of smoking per day (never, ever, current and <0.5 pack/day, current and ≥0.5–1 pack/day, or current and ≥1 pack/day), year of smoking cessation, physical activity (none, light, moderate, or heavy), height (grouped by quartiles), BMI (<18.5, 18.5–22.9, 23.0–24.9, or ≥25), systolic and diastolic blood pressure, total cholesterol, and fasting blood and urine glucose levels. More in-depth descriptions of the rationale of the categorization of these variables were published previously [10, 11]. Three different model selection processes (forward, backward, stepwise) were employed in the multivariable analysis using α = 0.10. Graphical checks for the proportional hazards assumption were done. Age and its quadratic term were also included in the model as risk factors to improve the model fit.

The probability of developing pancreatic cancer within 8 years (t = 8) for an individual with K risk factors is estimated as follows:

$$P(pancreatic\;cancer)=1-{\left[S_{0}(t)\right]}^{\text{ exp}\left(f(x)\right)},$$

where *f*(*x*) = *β*_*1*_(*x*_*1*_−*M*_*1*_)+*β*_*2*_(*x*_*2*_−*M*_*2*_)+…+*β*_*k*_(*x*_*K*_−*M*_*K*_), *β*_*1*_,…,*β*_*K*_ are Cox proportional hazard model coefficients, *x*_*1*_,…,*x*_*K*_ are the values of K risk factors at baseline, and *M*_*1*_,…,*M*_*K*_ are the average values of corresponding risk factors. Baseline survival probability *S*_0_(*t*) indicates the survival probability at time t for an individual at time t = 8 whose covariate values are equal to the mean value of each risk factor. The detailed procedures can be found in the S1 Appendix.

### Model validation

An independent population was used for external validation of the developed models, which allowed us to evaluate the performance of the models with respect to discrimination and calibration.

Discrimination is a model’s ability to distinguish between non-events and events. This can be quantified by calculating the C-statistic for the survival model developed by Nam [12]. The C-statistic is a concordance measure analogous to the receiver operating characteristic (ROC) curve area for the logistic model [13]. The value indicates the probability that a model produces higher risks for those who develop pancreatic cancer within 8 years of follow-up compared to those who do not develop pancreatic cancer [13]. An SAS macro was used to calculate the C-statistic with 95% confidence intervals (CIs).

Calibration measures how closely the predicted probabilities agree numerically with the actual outcomes. We used a Hosmer-Lemeshow (H-L) type χ^2^ statistic developed by Nam [12]. This χ^2^ statistic was calculated by dividing the data into 10 groups (deciles) based on the predicted probabilities produced by the model in ascending order. Then, for each decile, the average predicted probabilities were compared to the actual risk probabilities estimated by the Kaplan-Meier approach [14].

All statistical analyses were performed using SAS, version 9.1 (SAS institute, Cary, NC) and Stata version 10 (StataCorp LP, College Station, TX).

## Results

### Cancer incidence and baseline characteristics

Among 1,289,933 men and 557,701 women, 1,634 (0.13%) men and 561 (0.10%) women were newly diagnosed with pancreatic cancer during 8 years of follow-up, and the incidence rates in men and women were 11.56 and 9.39 cases per 100,000 person-years, respectively (Table 1). Across age groups, the incidence proportions for men were highest in the 50s followed by the 60s, 40s, and 70s. In women, the incidence proportions were higher in their 60s, followed by 50s and 70s.

**Table 1:** Pancreatic cancer incidence rates.

Age	Men					Women				
	Study cohort				Total Korean population	Study cohort				Total Korean population
	Total number	Person -years (pyrs)	Newly diagnosed cancer cases	Cancer incidence rate (/100,000 pyrs)	Cancer incidence rate^a^ (/100,000 pyrs)	Total number	Person -years (pyrs)	Newly diagnosed cancer cases	Cancer incidence rate (/100,000 pyrs)	Cancer incidence rate^a^ (/100,000 pyrs)
30–34	234,519	2,652,235	34	1.3	0.4	60,956	697,530	3	0.4	0.2
35–39	248,293	2,800,831	70	2.5	1.2	59,209	667,603	9	1.4	1.1
40–44	242,177	2,695,963	147	5.5	3.1	116,002	1,254,254	32	2.6	1.5
45–49	177,027	1,959,249	204	10.4	5.9	81,527	880,240	45	5.1	3.4
50–54	147,133	1,604,463	319	19.9	12.0	71,236	761,365	72	9.5	5.2
55–59	118,020	1,255,300	331	26.4	20.5	63,551	669,763	91	13.6	10.1
60–64	70,513	715,224	291	40.7	32.8	50,249	517,627	119	23.0	16.1
65–69	28,146	261,363	119	45.5	48.7	29,811	296,694	98	33.0	26.3
70–74	16,372	138,059	84	60.8	66.5	17,258	161,408	65	40.3	42.9
75–80	7,733	57,171	35	61.2	96.0^b^	7,902	65,723	27	41.1	58.5^b^
Total	1,289,933	14,139,858	1,634	11.56	-	557,701	5,972,207	561	9.39	-

^a^ Ministry of Health and Welfare.Annual report of cancer incidence (2005) and survival (1993–2005) in Korea, 2008.

^b^ Incidence rates for men and women aged 75–79 years.

Tables 2 and 3 provide the baseline characteristics and age-adjusted results of univariate analyses for each risk factor in men and women. The mean (standard deviation) ages of the men and women were 44.6 (10.33) years and 48.6 (11.27) years, respectively.

**Table 2:** Baseline characteristics, and univariate and multivariable analyses in men.

Risk factor	Frequency		Univariate^a^		Multivariable		
	Total (%) (n = 1,289,933)	Event (%) (n = 1,634)	HR^c^ (95% CI)	P-value	β	HR^c^ (95% CI)	P-value
Age-Mean_age_, years					**0.1344**	**1.14 (1.13 to 1.16)**	**<0.001**
(Age-Mean_age_)^2^, years^2^					**-0.0018**	**1.00 (1.00 to 1.00)**	**<0.001**
Height (cm)							
≤165	388,485 (30.12)	632 (38.68)	1.00 (Ref.)			1.00 (Ref.)	
>165, ≤168	247,472 (19.18)	329 (20.13)	1.10 (0.96 to 1.26)	0.171	0.0694	1.07 (0.94 to 1.23)	0.311
>168, ≤172	355,330 (27.55)	413 (25.28)	**1.17 (1.03 to 1.33)**	**0.015**	**0.1415**	**1.15 (1.02 to 1.31)**	**0.028**
>172	298,646 (23.15)	260 (15.91)	1.08 (0.93 to 1.26)	0.297	0.0881	1.09 (0.94 to 1.27)	0.245
BMI (kg/m^2^)							
<18.5	30,865 (2.39)	49 (3.00)	0.98 (0.73 to 1.31)	0.869	0.0128	1.01 (0.76 to 1.36)	0.932
18.5–22.9	527,268 (40.88)	628 (38.43)	1.00 (Ref.)			1.00 (Ref.)	
23.0–24.9	365,626 (28.34)	493 (30.17)	**1.16 (1.03 to 1.30)**	**0.016**	**0.1447**	**1.16 (1.03 to 1.30)**	**0.017**
≥25.0	366,174 (28.39)	464 (28.40)	**1.12 (1.00 to 1.27)**	**0.057**	**0.1176**	**1.13 (1.00 to 1.27)**	**0.058**
Blood glucose (mg/dL)							
<140	1,236,490 (95.86)	1,498 (91.68)	1.00 (Ref.)			1.00 (Ref.)	
≥140	53,443 (4.14)	136 (8.32)	**1.50 (1.26 to 1.79)**	**<0.001**	**0.2364**	**1.27 (1.02 to 1.58)**	**0.033**
Urine glucose							
Negative	1,237,536 (95.94)	1,504 (92.04)	1.00 (Ref.)			1.00 (Ref.)	
Positive	52,397 (4.06)	130 (7.96)	**1.49 (1.24 to 1.78)**	**<0.001**	**0.2018**	**1.22 (0.98 to 1.53)**	**0.075**
Cholesterol (mg/dL)							
≤200	821,613 (63.69)	978 (59.85)	1.00 (Ref.)				
201–239	347,321 (26.93)	480 (29.38)	1.06 (0.95 to 1.18)	0.287			
≥240	120,999 (9.38)	176 (10.77)	1.08 (0.92 to 1.27)	0.364			
Smoking							
Never	377,721 (29.28)	408 (24.97)	1.00 (Ref.)			1.00 (Ref.)	
Past	174,710 (13.54)	220 (13.46)	**1.16 (0.98 to 1.37)**	**0.078**	0.1355	1.15 (0.97 to 1.35)	0.106
Current, <0.5 pack/day	118,383 (9.18)	157 (9.61)	**1.28 (1.06 to 1.53)**	**0.010**	**0.2702**	**1.31 (1.09 to 1.58)**	**0.004**
Current, 0.5–0.9 pack/day	436,730 (33.86)	576 (35.25)	**1.68 (1.48 to 1.91)**	**<0.001**	**0.5012**	**1.65 (1.45 to 1.88)**	**<0.001**
Current, ≥1 pack/day	182,389 (14.14)	273 (16.71)	**2.09 (1.79 to 2.43)**	**<0.001**	**0.6966**	**2.01 (1.71 to 2.36)**	**<0.001**
Age at smoking initiation^b^							
Never	377,721 (29.28)	408 (24.97)	**0.61 (0.54 to 0.68)**	**<0.001**			
Past	174,710 (13.54)	220 (13.46)	**0.70 (0.61 to 0.82)**	**<0.001**			
Age≥25	480,602 (37.26)	797 (48.78)	1.00 (Ref.)			1.00 (Ref.)	
Age<25	256,900 (19.92)	209 (12.79)	1.10 (0.94 to 1.30)	0.232	0.1134	1.12 (0.95 to 1.32)	0.170
Drinking habit							
≤2–3 times/month	645,880 (50.07)	839 (51.35)	1.00 (Ref.)				
≥1–2 times/week	644,053 (49.93)	795 (48.65)	**1.09 (0.99 to 1.20)**	**0.089**			
Meal preferences							
Vegetables	261,224 (20.25)	306 (18.73)	1.00 (Ref.)				
Vegetables/meat	918,130 (71.18)	1,186 (72.58)	**1.16 (1.02 to 1.32)**	**0.020**			
Meat	108,092 (8.38)	136 (8.32)	1.14 (0.93 to 1.40)	0.200			
No response	2,487 (0.19)	6 (0.37)	1.85 (0.82 to 4.14)	0.137			
Frequency of meat intake							
≤1 time/week	591,704 (45.87)	725 (44.37)	1.00 (Ref.)				
2–3 times/week	630,714 (48.90)	774 (47.37)	1.08 (0.97 to 1.19)	0.157			
≥4 times/week	67,515 (5.23)	135 (8.26)	1.14 (0.95 to 1.38)	0.159			
Eating habits							
Bland	206,192 (15.98)	240 (14.69)	1.00 (Ref.)				
Medium	806,217 (62.50)	1,005 (61.51)	1.12 (0.98 to 1.29)	0.108			
Spicy or salty	275,606 (21.37)	388 (23.75)	**1.25 (1.06 to 1.47)**	**0.007**			
No response	1,918 (0.15)	1 (0.06)	0.39 (0.06 to 2.81)	0.352			

^a^ univariate analyses results adjusted for age.

^b^ only current smoking was used in multivariable analysis.

^c^ hazard ratio.

Bold font indicates statistically significant at α = 0.10.

**Table 3:** Baseline characteristics, and univariate and multivariable analyses in women.

Risk factor	Frequency		Univariate^a^		Multivariable		
	Total (%) (n = 557,701)	Event (%) (n = 561)	HR^b^ (95% CI)	P-value	β	HR^b^ (95% CI)	P-value
Age-Mean_age_, years					**0.1181**	**1.13 (1.11 to 1.14)**	**<0.001**
(Age-Mean_age_)^2^, years^2^					**-0.0017**	**1.00 (1.00 to 1.00)**	**<0.001**
Height (cm)							
≤151	148,585 (26.64)	248 (44.21)	1.00 (Ref.)			1.00 (Ref.)	
>151, ≤155	147,694 (26.48)	149 (26.56)	1.03 (0.84 to 1.27)	0.781	-0.0117	0.99 (0.80 to 1.22)	0.913
>155, ≤158	112,895 (20.24)	70 (12.48)	0.83 (0.63 to 1.10)	0.195	-0.2127	0.81 (0.62 to 1.06)	0.128
>158	148,527 (26.63)	94 (16.76)	1.14 (0.88 to 1.48)	0.327	0.1508	1.16 (0.90 to 1.50)	0.247
BMI (kg/m^2^)							
<18.5	23,277 (4.17)	24 (4.28)	1.40 (0.91 to 2.14)	0.126	0.3405	1.41 (0.91 to 2.16)	0.121
18.5–22.9	246,056 (44.12)	169 (30.12)	1.00 (Ref.)			1.00 (Ref.)	
23.0–24.9	132,528 (23.76)	141 (25.13)	**1.27 (1.01 to 1.59)**	**0.038**	**0.1997**	**1.22 (0.98 to 1.53)**	**0.082**
≥25.0	155,840 (27.94)	227 (40.46)	**1.48 (1.21 to 1.81)**	**<0.001**	**0.3390**	**1.40 (1.15 to 1.72)**	**0.001**
Blood glucose (mg/dL)							
<140	538,848 (96.62)	522 (93.05)	1.00 (Ref.)			1.00 (Ref.)	
≥140	18,853 (3.38)	39 (6.95)	**1.36 (0.98 to 1.88)**	**0.066**	0.0747	1.08 (0.72 to 1.62)	0.719
Urine glucose							
Negative	544,690 (97.67)	531 (94.65)	1.00 (Ref.)			1.00 (Ref.)	
Positive	13,011 (2.33)	30 (5.35)	**1.59 (1.10 to 2.30)**	**0.014**	0.3749	1.46 (0.92 to 2.30)	0.110
Cholesterol (mg/dL)							
≤200	339,912 (60.95)	273 (48.66)	1.00 (Ref.)				
201–239	152,851 (27.41)	190 (33.87)	1.08 (0.89 to 1.30)	0.438			
≥240	64,938 (11.64)	98 (17.47)	1.10 (0.87 to 1.39)	0.429			
Smoking							
Never	529,318 (94.91)	483 (86.10)	1.00 (Ref.)			1.00 (Ref.)	
Past	5,884 (1.06)	11 (1.96)	1.07 (0.59 to 1.95)	0.832	0.1154	1.12 (0.62 to 2.05)	0.707
Current	22,499 (4.03)	67 (11.94)	**1.74 (1.34 to 2.26)**	**<0.001**	**0.5863**	**1.80 (1.38 to 2.34)**	**<0.001**
Age at smoking initiation							
Never	529,318 (94.91)	483 (86.10)	**0.57 (0.44 to 0.75)**	**<0.001**			
Past	5,884 (1.06)	11 (1.96)	0.61 (0.32 to 1.16)	0.133			
Age≥25	21,445 (3.85)	66 (11.76)	1.00 (Ref.)				
Age<25	1,054 (0.19)	1 (0.18)	1.04 (0.14 to 7.52)	0.970			
Drinking habit							
≤2–3 times/month	525,092 (94.15)	518 (92.34)	1.00 (Ref.)			1.00 (Ref.)	
≥1–2 times/week	32,609 (5.85)	43 (7.66)	1.25 (0.92 to 1.71)	0.156	0.1481	1.16 (0.85 to 1.59)	0.358
Meal preferences							
Vegetables	192,896 (34.59)	226 (40.29)	1.00 (Ref.)				
Vegetables/meat	338,423 (60.68)	316 (56.33)	1.02 (0.86 to 1.21)	0.842			
Meat	25,389 (4.55)	18 (3.21)	0.80 (0.49 to 1.29)	0.359			
No response	993 (0.18)	1 (0.18)	0.88 (0.12 to 6.29)	0.900			
Frequency of meat intake							
≤1 time/week	316,043 (56.67)	327 (58.29)	1.00 (Ref.)				
2–3 times/week	204,784 (36.72)	197 (35.12)	1.02 (0.85 to 1.22)	0.834			
≥4 times/week	36,874 (6.61)	37 (6.60)	0.90 (0.64 to 1.26)	0.529			
Eating habits							
Bland	90,712 (16.27)	97 (17.29)	1.00 (Ref.)				
Medium	371,647 (66.64)	360 (64.17)	1.01 (0.81 to 1.27)	0.914			
Spicy or salty	94,483 (16.94)	103 (18.36)	0.99 (0.75 to 1.31)	0.948			
No response	859 (0.15)	1 (0.18)	1.00 (0.14 to 7.14)	0.997			

^a^ univariate analyses results adjusted for age.

^b^ hazard ratio.

Bold font indicates statistically significant at α = 0.10.

### Risk factors and relative risk

The age-adjusted univariate analyses in men showed that height, BMI, blood glucose, urine glucose, smoking, meal preference (vegetables/meat), and eating habits (spicy or salty) are significant risk factors for pancreatic cancer. In the risk prediction model for men, age, height, BMI, blood glucose, urine glucose, smoking, and age at smoking initiation were finally included based on stepwise selection. To improve the model’s fit, we included a quadratic term of age (age^2^) and combined smoking status and the average value for the amount smoked per day into one variable termed “smoking” that comprised five categories: never, past, current <0.5 pack/day, current 0.5–0.99 pack/day, and current ≥1 pack/day. The pancreatic cancer risk increased as height and BMI increased. Higher blood glucose (≥140 mg/dL) was associated with an almost 30% increased risk compared to lower blood glucose (<140 mg/dL). Current smokers consuming more than 1 pack of cigarettes per day had double the risk compared to never smokers (Table 2).

For women, BMI, urine glucose, and current smoking were significant risk factors in the age-adjusted univariate analyses. In multivariable Cox regression model for women, age and its quadratic term (age^2^), height, BMI, blood glucose, urine glucose, and drinking habit were finally selected as factors. BMI greater than 25.0 was associated with a 1.4-fold increased risk compared to the normal range (18.5–22.9). Current smokers also had a 1.8-fold higher risk than never smokers (Table 3).

### Validation of the risk prediction models

The risk prediction models were validated in an independent cohort by evaluating their discrimination and calibration abilities with respect to the C-statistic and the H-L type χ^2^ statistic. As shown in Table 4, baseline characteristics for the validation cohort were similar to those in the derivation cohort. The incidence rates for men aged more than 60 years in the validation set slightly increased compared to the derivation set because of the higher proportion of older participants (S1 Table).

**Table 4:** Baseline characteristics in the validation cohort.

Risk factor	Men		Women	
	Total (%) (n = 500,046)	Event (%) (n = 711)	Total (%) (n = 627,629)	Event (%) (n = 576)
Height (cm) in men				
≤165	178,435 (35.68)	356 (50.07)		
>165, ≤168	95,627 (19.12)	136 (19.13)		
>168, ≤172	119,951 (23.99)	137 (19.27)		
>172	106,033 (21.20)	82 (11.53)		
Height (cm) in women				
≤151			181,483 (28.92)	293 (50.87)
>151, ≤155			164,080 (26.14)	148 (25.69)
>155, ≤158			121,705 (19.39)	74 (12.85)
>158			160,361 (25.55)	61 (10.59)
BMI (kg/m^2^)				
<18.5	15,641 (3.13)	40 (5.63)	25,726 (4.10)	15 (2.60)
18.5–22.9	198,824 (39.76)	319 (44.87)	274,568 (43.75)	203 (35.24)
23.0–24.9	132,473 (26.49)	174 (24.47)	148,049 (23.59)	144 (25.00)
≥25.0	153,108 (30.62)	178 (25.04)	179,286 (28.57)	214 (37.15)
Blood glucose (mg/dL)				
<140	474,825 (94.96)	633 (89.03)	605,146 (96.42)	527 (91.49)
≥140	25,221 (5.04)	78 (10.97)	22,483 (3.58)	49 (8.51)
Urine glucose				
Negative	476,949 (95.38)	649 (91.28)	613,231 (97.71)	544 (94.44)
Positive	23,097 (4.62)	62 (8.72)	14,398 (2.29)	32 (5.56)
Smoking				
Never	160,044 (32.01)	231 (32.49)	599,007 (95.44)	510 (88.54)
Past	61,255 (12.25)	92 (12.94)	6,480 (1.03)	9 (1.56)
Current			22,142 (3.53)	57 (9.90)
<0.5 pack/day	49,696 (9.94)	87 (12.24)		
0.5–0.9 pack/day	147,971 (29.59)	196 (27.57)		
≥1 pack/day	81,080 (16.21)	105 (14.77)		
Age at smoking initiation				
Never	160,044 (32.01)	231 (32.49)		
Past	61,255 (12.25)	92 (12.94)		
Age≥25	179,459 (35.89)	329 (46.27)		
Age<25	99,288 (19.86)	59 (8.30)		
Drinking habit				
≤2–3 times/month			588,165 (93.71)	545 (94.62)
≥1–2 times/week			39,646 (6.29)	31 (5.38)

**Fig 1:**
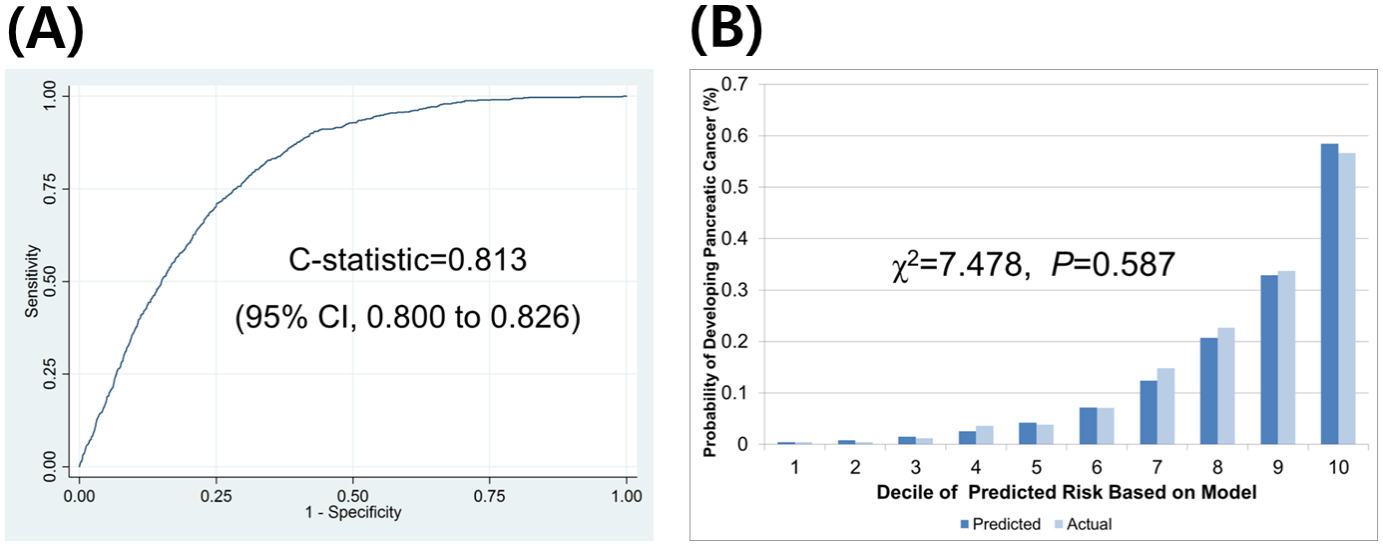
Discrimination and calibration ability of the pancreatic cancer prediction model in men as assessed in an external validation set (n = 500,046, events = 711). (A) discrimination ability and (B) calibration ability.

**Fig 2:**
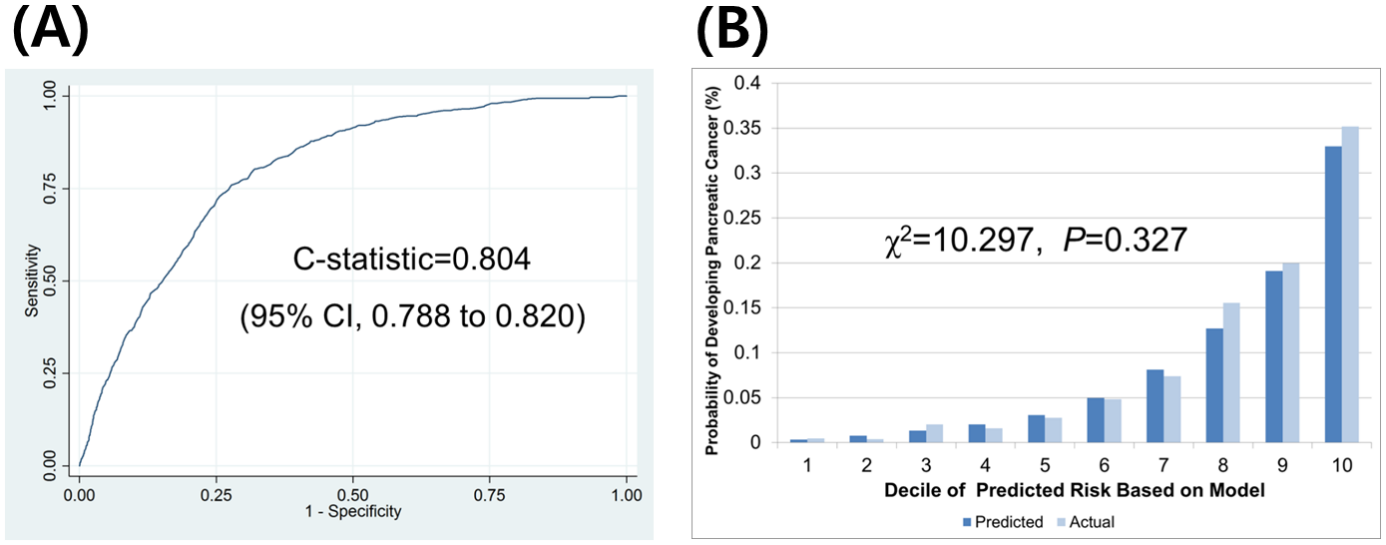
Discrimination and calibration ability of the pancreatic cancer prediction model in women as assessed in an external validation set (n = 627,629, events = 576). (A) discrimination ability and (B) calibration ability.

The C-statistics (95% CI) were 0.813 (0.800 to 0.826) for men and 0.804 (0.788 to 0.820) for women, respectively. The H-L type χ^2^ statistics were 7.478 (p = 0.587) and 10.297 (p = 0.327) for men and women, respectively. The validation results are provided in Figs 1 and 2.

### Illustration of individual absolute risk estimation for pancreatic cancer

The absolute risk estimates for pancreatic cancer within 8 years are illustrated in S2 and S3 Tables for men and women, respectively. In S2 Table, no. 3 is a 50-year-old man with a height of ≤165 cm, BMI of 18.5–22.9, negative urine glucose, <140 mg/dL blood glucose, and classified as a non-smoker. His risk of pancreatic cancer within 8-years was only 0.0557%. On the other hand, no. 1 is a 50-year-old man who was between 168 and 172 cm in height, with a BMI of 23.0–24.9, positive urine glucose, and is a current smoker consuming ≥1 pack/day, started smoking at 25 years of age, and has ≥140 mg/dL blood glucose. This patient had a 0.2579% risk, which was 4.6 times greater than that of no.3. In the same manner, we can interpret that no.16, who had the same risk profile as no.1 but was 25 years older, has an approximately 6-times greater risk than no. 1 (1.5117% vs. 0.0557%).

For women, 8-year risk estimates are described in S3 Table for each risk profile. If a woman is 75 years old, >158 cm in height, with a BMI of <18.5, no urine glucose, is a current smoker, drinks ≥1–2 times per week, and has a ≥140 mg/dL blood glucose, then she has a 1.1952% absolute risk of pancreatic cancer over 8 years, which is roughly 6.6-times greater risk than a woman of the same age who is between 155 and 158 cm in height with healthier physical conditions and smoking and drinking habits, such as no.18 (1.1952% vs. 0.1819%)

### Cumulative incidence probabilities of five risk groups

**Fig 3:**
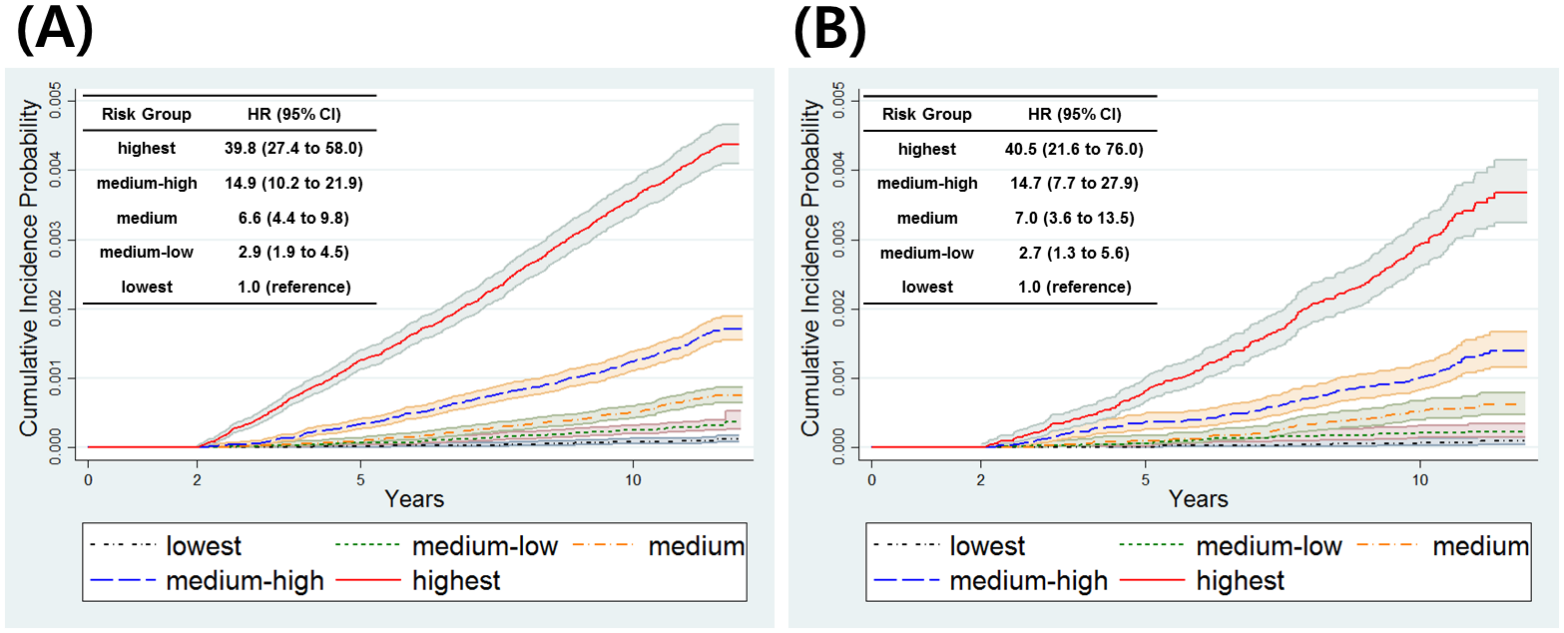
Cumulative incidence probability for five risk groups. (A) men and (B) women.

We divided derivation sets of men and women into five risk groups based on quintiles of the estimated probability of developing pancreatic cancer. The cumulative incidence probability and hazard ratio for each risk group is provided in Fig 3. At 10 years, the cumulative incidence probabilities of the highest risk group were 0.359% (95% CI: 0.335–0.384) and 0.292% (95% CI: 0.261–0.327) in men and women, respectively, and those of the lowest risk group were 0.009% (95% CI: 0.006–0.013) and 0.006% (95% CI: 0.003–0.013) in men and women, respectively. Medium-low, medium, medium-high, and highest risk groups had significantly higher hazard ratios than the corresponding lowest risk group in both men and women.

## Discussion

Despite improvements in the clinical management of pancreatic cancer, limited advances have been made in the early detection of this highly lethal malignancy [15]. Within the population of resectable pancreatic cancer patients, 5-year survival exceeds 75% in the subset with well-differentiated stage I cancers < 1 cm [5]. Thus, early detection of pancreatic cancer, or even precursor lesions, is the most intuitive approach for improving the overall prognosis of this lethal and frustrating cancer. Because of the overall low prevalence of pancreatic cancer in the general population, current screening efforts are mainly directed at populations at high risk of developing pancreatic cancer. The present risk prediction models were developed to guide healthcare professionals and individuals in their decision making regarding further screening efforts and lifestyle changes in order to inform individuals about their risks of having pancreatic cancer. This approach is meant to supplement the reasoning and decision making of healthcare professionals by providing more objectively estimated probabilities [16].

However, very few studies in the literature have involved a prediction model for pancreatic cancer. One previous study proposed a method that combines PubMed knowledge and electronic health records to develop a weighted Bayesian Network Inference model for pancreatic cancer prediction [17]. Though pancreatic cancer was used as a sample disease in the initial study, clinical implementation remains a challenge. The etiology of pancreatic cancer remains to be established, but several known genetic and environmental factors have been associated with its development. Thus far, risk factors accounting for up to 30% of the disease have been determined [18]. Among the few risk factors identified to date, cigarette smoking is the most consistent [19]. However, inconsistencies in the patterns of cigarette smoking and incidence between different countries, as well as the low relative risk, suggest that the disease is only partially attributable (~20%) to smoking [20]. Diabetes mellitus [21] and chronic pancreatitis [22] are additional predisposing factors for pancreatic cancer, but diabetes as a result of pancreatic cancer is not infrequent and chronic pancreatitis explains less than 3% of pancreatic cancer cases. In contrast, obesity has been reported to be associated with an approximate 20% increase in pancreatic cancer risk compared to normal weight [23]. In the present study, pancreatic cancer risk increased with increasing height or BMI. In the model for men, higher blood glucose levels (>140 mg/dL) were associated with an almost 30% increased risk of pancreatic cancer compared to lower blood glucose levels (<140 mg/dL). Unfortunately, the association between chronic pancreatitis and pancreatic cancer could not be investigated in these cohorts.

Alcohol consumption is a well-known risk factor for type II diabetes mellitus and chronic pancreatitis, both of which are associated with an increased risk of pancreatic cancer. However, the relationship between alcohol intake and pancreatic cancer risk has been too inconsistent to reach a conclusion on the association between alcohol intake and the risk of pancreatic cancer. A pooled analysis of the primary data from 14 prospective cohort studies [24] revealed a positive association of pancreatic cancer risk with alcohol intake, but it was only significant among women. In the present study, drinking habit was selected for the model for women only.

Thus far, no such early detection method has had sufficient sensitivity and specificity to serve as a tool for pancreatic cancer screening. In addition, the feasibility of pancreatic cancer screening among the general population is questionable owing to the overall low prevalence. Thus, existing research of screening has been restricted to high-risk individuals, such as those with Peutz-Jeghers syndrome, familial breast-ovarian cancer patients, and relatives of patients with familial pancreatic cancer with at least one affected first-degree relative [25]. The largest prospective study showed that screening asymptomatic high-risk individuals can detect pancreatic lesions, including curable, non-invasive high-grade neoplasms. However, such individuals with genetic syndromes account for less than 5% of all pancreatic cancer. The present study provides a unique opportunity to filter the population into high-risk individuals using a predictive model of the individual risk of sporadic pancreatic cancer.

Despite the large sample size, a central limitation of this cohort study is its retrospective nature, as it utilized existing subject data that are usually documented for other reasons and can address longer follow-up times, but usually at the expense of poorer, less systematically obtained data [26]. We used an independent population for model validation, but it was also from the same National Health Insurance data. Hence we should be more careful in generalizing these results. However, these risk prediction models for pancreatic cancer exhibited very good performance so that it can be a valuable tool for screening high risk individuals for pancreatic cancer.

Another limitation in this study is death due to other reasons than pancreatic cancer was considered as ‘censored’ rather than a competing risk of pancreatic cancer occurrence. However, we could find some publications for development of the risk prediction model for pancreatic cancer using similar censoring scheme for death [27–29] and there are already several publications where we used the same approach as in this study [11, 30, 31].

Since this model is based on Korean population, for Asian population, further studies will be needed for other races.

## Supporting Information

S1 Appendix Risk prediction of developing pancreatic cancer within 8 years. (DOCX)

S1 Table Pancreatic cancer incidence rates in the validation set. (DOCX)

S2 Table Eight-year absolute risk estimates of pancreatic cancer in men with different factor profiles. (DOCX)

S3 Table Eight-year absolute risk estimates of pancreatic cancer in women with different factor profiles. (DOCX)
