# Supplementary material for: Development and Validation of a Prediction Model to Estimate Individual Risk of Pancreatic Cancer
Source: PLoS One. 2016 Jan 11;11(1):e0146473. doi: 10.1371/journal.pone.0146473 (PMC4708985; doi:10.1371/journal.pone.0146473)
Supplement: S1 Appendix — (DOCX) [file pone.0146473.s001.docx]

S1 Appendix. Risk prediction of developing pancreatic cancer within 8 years

A. Application of tables for men

Step 1: Form a linear equation (A) using the β-coefficient estimates

| A = 0.1344*[(Age-Mean_age_) – 0]  – 0.0018*[(Age-Mean_age_)^2^ – 106.6071]  + 0.0*(Height)  + 0.0694*(Height – 0.1918)  + 0.1415*(Height – 0.2755)  + 0.0881*(Height – 0.2315)  + 0.0128*(BMI – 0.0239)  + 0.0*(BMI)  + 0.1447*(BMI – 0.2834)  + 0.1176*(BMI – 0.2839)  + 0.0*(Urine glucose)  + 0.2018*(Urine glucose – 0.0406)  + 0.0*(Smoke)  + 0.1355*(Smoke – 0.1354)  + 0.2702*(Smoke – 0.0918)  + 0.5012*(Smoke – 0.3386)  + 0.6966*(Smoke – 0.1414)  + 0.0*(ASI)  + 0.1134*(ASI – 0.1992),  + 0.0*(Blood glucose)  + 0.2364*(Blood glucose – 0.0414) | if Height, ≤165 cm  if Height, >165 cm, ≤168 cm  if Height, >168 cm, ≤172 cm  if Height, >172 cm  if BMI, <18.5  if BMI, 18.5-22.9  if BMI, 23.0-24.9  if BMI, ≥25.0  if Urine glucose, (–)  if Urine glucose, (+)  if Never smoker  if Past smoker  if Current smoker, < 0.5 pack/day  if Current smoker, 0.5-0.99 pack/day  if Current smoker, ≥ 1 pack/day  if Age at smoking initiation, ≥ 25  if Age at smoking initiation, < 25  if Blood glucose, <140 mg/dL  if Blood glucose, ≥140 mg/dL |
| --- | --- |

Step 2: Exponential A, call it E

E= exp(A)

Step 3: Calculate the probability P=1 – S(t|t=8)^E^

Where S(t|t=8) is the survival probability estimate for the mean values of the risk factors in the model. Here, S(t|t=8) = 0.9996132043.

Consider a 50-year-old man with a current smoking habit of 1 pack/day, age at smoking initiation of 22 years, height of 170 cm, BMI of 23 kg/m^2^, positive urine glucose, and a blood glucose of 141 mg/dL.

A = 0.1344*[(50 – 45) – 0] – 0.0018*[25 – 106.6071]

+ 0.0694*(0 – 0.1918) + 0.1415*(1 – 0.2755) + 0.0881*(0 – 0.2315)

+ 0.0128*(0 – 0.0239) + 0.1447*(1 – 0.2834) + 0.1176*(0 – 0.2839)

+ 0.2018*(1 – 0.0406)

+ 0.1355*(0 – 0.1354) + 0.2702*(0 – 0.0918) + 0.5012*(0 – 0.3386) + 0.6966*(1 – 0.1414)

+ 0.1134*(1 – 0.1992)

+ 0.2364*(1 – 0.0414)

= 1.85398

E = exp(A) = exp(1.85398) = 6.38516

P = 1– S(t|t=8)^E^ = 1 – 0.9996132043^(6.38516)^ = 0.002467, for 0.2467% chance of developing pancreatic cancer over 8 years.

B. Application of tables for women

Step 1: Form a linear equation (A) using the β-coefficient estimates

| A = 0.1181*[(Age-Mean_age_) – 0]  – 0.0017*[(Age-Mean_age_)^2^ – 127.0656]  + 0.0*(Height)  – 0.0117*(Height – 0.2648)  – 0.2127*(Height – 0.2024)  + 0.1508*(Height – 0.2663)  + 0.3405*(BMI – 0.0417)  + 0.0*(BMI)  + 0.1997*(BMI – 0.2376)  + 0.3390*(BMI – 0.2794)  + 0.0*(Urine glucose)  + 0.3749*(Urine glucose – 0.0233)  + 0.0*(Smoke)  + 0.1154*(Smoke – 0.0106)  + 0.5863*(Smoke – 0.0403)  + 0.0*(Alcohol)  + 0.1481*(Alcohol – 0.0585)  + 0.0*(Blood glucose)  + 0.0747*(Blood glucose – 0.0338) | if Height, ≤151 cm  if Height, >151 cm, ≤155 cm  if Height, >155 cm, ≤158 cm  if Height, >158 cm  if BMI, <18.5  if BMI, 18.5-22.9  if BMI, 23.0-24.9  if BMI, ≥25.0  if Urine glucose, (–)  if Urine glucose, (+)  if Cigarette smoking habit, Never  if Cigarette smoking habit, Past  if Cigarette smoking habit, Current  if Alcohol consumption frequency,  ≤2-3 times/month  if Alcohol consumption frequency,  ≥1-2 times/week  if Blood glucose, <140 mg/dL  if Blood glucose, ≥140 mg/dL |
| --- | --- |

Step 2: Exponential A, call it E

E= exp(A)

Step 3: Calculate the probability P=1 – S(t|t=8)^E^

Where S(t|t=8) is the survival probability estimate for the mean values of the risk factors in the model. Here, S(t|t=8) = 0.9996882437.

Consider a 50-year-old female who currently smokes, with a height of 160 cm, BMI of 18 kg/m^2^, positive urine glucose, alcohol consumption frequency of 3 times/week, and blood glucose of 141 mg/dL.

A = 0.1181*[(50 – 49) – 0] – 0.0017*[1 – 127.0656]

– 0.0117*(0 – 0.2648) – 0.2127*(0 – 0.2024) + 0.1508*(1 – 0.2663)

+ 0.3405*(1 – 0.0417) + 0.1997*(0 – 0.2376) + 0.3390*(0 – 0.2794)

+ 0.3749*(1 – 0.0233)

+ 0.1154*(0 – 0.0106) + 0.5863*(1 – 0.0403)

+ 0.1481*(1 – 0.0585)

+ 0.0747*(1 – 0.0338)

= 1.812563

E = exp(A) = exp(1.812563) = 6.126128

P = 1– S(t|t=8)^E^ = 1 – 0.9996882437^(6.126128)^ = 0.001908, for 0.1908% chance of developing pancreatic cancer over 8 years.
