# Supplementary material for: Development and Validation of a Prediction Model to Estimate Individual Risk of Pancreatic Cancer
Source: PLoS One. 2016 Jan 11;11(1):e0146473. doi: 10.1371/journal.pone.0146473 (PMC4708985; doi:10.1371/journal.pone.0146473)
Supplement: S1 Table — (DOCX) [file pone.0146473.s002.docx]

S2 Table. Pancreatic cancer incidence rates in the validation set

| Age | Men | | | | | Women | | | | |
| --- | --- | --- | --- | --- | --- | --- | --- | --- | --- | --- |
|  | Study cohort | | | | Total  Korean  population | Study cohort | | | | Total  Korean  population |
|  | Total  number | Person-  years  (pyrs) | Newly  diagnosed  cancer cases | Cancer  incidence  rate  (/100,000pyrs) | Cancer  incidence  rate^a^  (/100,000 pyrs) | Total  number | Person-  years  (pyrs) | Newly  diagnosed  cancer  cases | Cancer  incidence  rate  (/100,000pyrs) | Cancer  incidence  rate^a^  (/100,000 pyrs) |
| 30–34 | 103,520 | 951,311 | 6 | 0.6 | 0.4 | 62,965 | 595,613 | 6 | 1.0 | 0.3 |
| 35–39 | 66,019 | 582,501 | 12 | 2.1 | 1.1 | 66,465 | 618,472 | 3 | 0.5 | 0.4 |
| 40–44 | 84,749 | 727,711 | 33 | 4.5 | 2.2 | 129,549 | 1,115,566 | 29 | 2.6 | 1.9 |
| 45–49 | 51,240 | 441,884 | 62 | 14.0 | 5.8 | 82,878 | 715,727 | 36 | 5.0 | 3.0 |
| 50–54 | 53,018 | 448,383 | 83 | 18.5 | 13.3 | 74,017 | 627,893 | 41 | 6.5 | 5.5 |
| 55–59 | 48,311 | 401,988 | 109 | 27.1 | 21.7 | 66,226 | 557,768 | 79 | 14.2 | 9.6 |
| 60–64 | 42,892 | 340,796 | 162 | 47.5 | 35.9 | 64,134 | 527,601 | 128 | 24.3 | 17.1 |
| 65–69 | 24,636 | 186,431 | 113 | 60.6 | 43.6 | 41,582 | 334,579 | 111 | 33.2 | 28.3 |
| 70–74 | 16,644 | 117,380 | 79 | 67.3 | 67.7 | 26,606 | 204,998 | 92 | 44.9 | 39.8 |
| 75–80 | 9,017 | 57,246 | 52 | 90.8 | 79.1^b^ | 13,207 | 94,578 | 51 | 53.9 | 56.1^b^ |
| Total | 500,046 | 4,255,631 | 711 | 16.7 |  | 627,629 | 5,392,794 | 576 | 10.7 |  |

^a^ Ministry of Health and Welfare. Annual report of cancer incidence (2007) and survival (1993–2007) in Korea, 2009.

^b^ Incidence rates for men and women aged 75–79 years.
