# Supplementary material for: Development and Validation of a Prediction Model to Estimate Individual Risk of Pancreatic Cancer
Source: PLoS One. 2016 Jan 11;11(1):e0146473. doi: 10.1371/journal.pone.0146473 (PMC4708985; doi:10.1371/journal.pone.0146473)
Supplement: S2 Table — (DOCX) [file pone.0146473.s003.docx]

S3 Table. Eight-year absolute risk estimates of pancreatic cancer in men with different factor profiles

| No. | Age  (y) | Height  (cm) | BMI | Urine  glucose | Smoking | Age at  smoking  initiation  (y) | Blood  glucose  (mg/dL) | 8-year absolute risk  (%) |
| --- | --- | --- | --- | --- | --- | --- | --- | --- |
| 1 | 50 | >168, ≤172 | 23.0-24.9 | positive | current, ≥1 pack | < 25 | ≥140 | 0.2579 |
| 2 | 50 | >165, ≤168 | 23.0-24.9 | negative | current, <0.5 pack | < 25 | <140 | 0.1012 |
| 3 | 50 | ≤165 | 18.5-22.9 | negative | non-smoking | – | <140 | 0.0557 |
| 4 | 55 | >168, ≤172 | 23.0-24.9 | positive | current, ≥1 pack | < 25 | ≥140 | 0.4387 |
| 5 | 55 | >165, ≤168 | 23.0-24.9 | negative | current, <0.5 pack | < 25 | <140 | 0.1722 |
| 6 | 55 | ≤165 | 18.5-22.9 | negative | non-smoking | – | <140 | 0.0947 |
| 7 | 60 | >168, ≤172 | 23.0-24.9 | positive | current, ≥1 pack | < 25 | ≥140 | 0.6826 |
| 8 | 60 | >165, ≤168 | 23.0-24.9 | negative | current, <0.5 pack | < 25 | <140 | 0.2681 |
| 9 | 60 | ≤165 | 18.5-22.9 | negative | non-smoking | – | <140 | 0.1475 |
| 10 | 65 | >168, ≤172 | 23.0-24.9 | positive | current, ≥1 pack | < 25 | ≥140 | 0.9720 |
| 11 | 65 | >165, ≤168 | 23.0-24.9 | negative | current, <0.5 pack | < 25 | <140 | 0.3821 |
| 12 | 65 | ≤165 | 18.5-22.9 | negative | non-smoking | – | <140 | 0.2103 |
| 13 | 70 | >168, ≤172 | 23.0-24.9 | positive | current, ≥1 pack | < 25 | ≥140 | 1.2669 |
| 14 | 70 | >165, ≤168 | 23.0-24.9 | negative | current, <0.5 pack | < 25 | <140 | 0.4984 |
| 15 | 70 | ≤165 | 18.5-22.9 | negative | no smoking | – | <140 | 0.2745 |
| 16 | 75 | >168, ≤172 | 23.0-24.9 | positive | current, ≥1 pack | < 25 | ≥140 | 1.5117 |
| 17 | 75 | >165, ≤168 | 23.0-24.9 | negative | current, <0.5 pack | < 25 | <140 | 0.5952 |
| 18 | 75 | ≤165 | 18.5-22.9 | negative | non-smoking | – | <140 | 0.3278 |
