# Supplementary material for: Development and Validation of a Prediction Model to Estimate Individual Risk of Pancreatic Cancer
Source: PLoS One. 2016 Jan 11;11(1):e0146473. doi: 10.1371/journal.pone.0146473 (PMC4708985; doi:10.1371/journal.pone.0146473)
Supplement: S3 Table — (DOCX) [file pone.0146473.s004.docx]

S4 Table. Eight-year absolute risk estimates of pancreatic cancer in women with different factor profiles

| No. | Age  (y) | Height  (cm) | BMI | Urine  glucose | Smoking | Drinking  habit | Blood  glucose (mg/dL) | 8-year absolute risk (%) |
| --- | --- | --- | --- | --- | --- | --- | --- | --- |
| 1 | 50 | >158 | <18.5 | positive | current | ≥1-2 times/week | ≥140 | 0.1988 |
| 2 | 50 | >151, ≤155 | 23.0-24.9 | negative | current | ≥1-2 times/week | <140 | 0.0937 |
| 3 | 50 | >155, ≤158 | 18.5-22.9 | negative | never | ≤2-3 times/month | <140 | 0.0301 |
| 4 | 55 | >158 | <18.5 | positive | current | ≥1-2 times/week | ≥140 | 0.3361 |
| 5 | 55 | >151, ≤155 | 23.0-24.9 | negative | current | ≥1-2 times/week | <140 | 0.1585 |
| 6 | 55 | >155, ≤158 | 18.5-22.9 | negative | never | ≤2-3 times/month | <140 | 0.0510 |
| 7 | 60 | >158 | <18.5 | positive | current | ≥1-2 times/week | ≥140 | 0.5228 |
| 8 | 60 | >151, ≤155 | 23.0-24.9 | negative | current | ≥1-2 times/week | <140 | 0.2466 |
| 9 | 60 | >155, ≤158 | 18.5-22.9 | negative | never | ≤2-3 times/month | <140 | 0.0793 |
| 10 | 65 | >158 | <18.5 | positive | current | ≥1-2 times/week | ≥140 | 0.7483 |
| 11 | 65 | >151, ≤155 | 23.0-24.9 | negative | current | ≥1-2 times/week | <140 | 0.3532 |
| 12 | 65 | >155, ≤158 | 18.5-22.9 | negative | never | ≤2-3 times/month | <140 | 0.1137 |
| 13 | 70 | >158 | <18.5 | positive | current | ≥1-2 times/week | ≥140 | 0.9857 |
| 14 | 70 | >151, ≤155 | 23.0-24.9 | negative | current | ≥1-2 times/week | <140 | 0.4655 |
| 15 | 70 | >155, ≤158 | 18.5-22.9 | negative | never | ≤2-3 times/month | <140 | 0.1499 |
| 16 | 75 | >158 | <18.5 | positive | current | ≥1-2 times/week | ≥140 | 1.1952 |
| 17 | 75 | >151, ≤155 | 23.0-24.9 | negative | current | ≥1-2 times/week | <140 | 0.5648 |
| 18 | 75 | >155, ≤158 | 18.5-22.9 | negative | never | ≤2-3 times/month | <140 | 0.1819 |
